# Cytomegalovirus serology in young to mid‐adult life and decline of lung function

**DOI:** 10.1111/crj.13600

**Published:** 2023-03-16

**Authors:** Raffaella Nenna, Jing Zhai, Amber Spangenberg, Duane L. Sherrill, Fernando D. Martinez, Marilyn Halonen, Stefano Guerra

**Affiliations:** ^1^ Asthma and Airway Disease Research Center University of Arizona Tucson Arizona USA; ^2^ Department of Maternal Infantile and Urological Sciences “Sapienza” University of Rome Rome Italy; ^3^ Mel and Enid Zuckerman College of Public Health University of Arizona Tucson Arizona USA; ^4^ Department of Medicine, College of Medicine – Tucson University of Arizona Tucson AZ USA

**Keywords:** airflow limitation, CMV, COPD, epidemiology, lung function

## Abstract

**Introduction:**

Cytomegalovirus (CMV) seropositivity has been recently linked to severity and progression of asthma, cystic fibrosis, and chronic obstructive pulmonary disease (COPD). To date, no longitudinal study has addressed the relation of CMV serology to levels and decline of lung function in the general adult population.

**Methods:**

We evaluated 403 participants from the Tucson Epidemiological Study of Airway Obstructive Disease (TESAOD) who at enrollment were aged 28–55 years and completed lung function tests. During follow‐up, the 403 participants completed on average 7.2 lung function tests per subject for a total of 2908 observations over a mean period of 14.7 years. We tested CMV serology in serum samples from enrollment and categorized participants into low, medium, and high CMV serology based on tertiles. The relation of CMV serology at enrollment to lung function levels and decline during follow‐up was tested in multivariate random coefficients models.

**Results:**

After full adjustment, participants in the highest CMV serology tertile had faster declines of forced expiratory volume in 1 s (FEV_1_) and FEV_1_/forced vital capacity (FVC) compared with subjects in the lowest tertile (by −7.9 ml/year 95% confidence interval [−13.9 ml/year, −1.93 ml/year], and by −0.13%/year [−0.23%/year, −0.026%/year], respectively). These CMV effects were additive with those of cigarette smoking. No associations were found between CMV serology and FVC, indicating specific effects of CMV seropositivity on airflow limitation.

**Conclusion:**

High CMV serology in young to mid‐adult life may be linked to increased COPD risk through an accelerated decline of lung function.

## Graphical abstract

Cytomegalovirus (CMV) seropositivity has been recently linked to severity and progression of obstructive lung diseases. Among 403 participants from the longitudinal, population‐based TESAOD study aged 28–55 years at enrollment, we found that, after full adjustment for covariates, high CMV serology at enrollment was associated with accelerated decline of FEV_1_ and FEV_1_/FVC (see figure) during follow‐up.

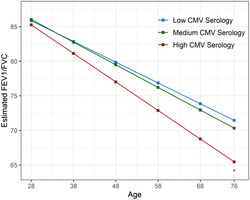

## 1 INTRODUCTION

Cytomegalovirus (CMV) is a ubiquitous virus that can persist in many tissues and establish a lifelong latent infection with periodic reactivations. CMV infection has been associated with immunosenescence and increased mortality risk in adults. Recently, several reports^1^, ^2^, ^3^, ^4^, ^5^ have indicated a possible involvement of CMV in the inception and progression of obstructive lung diseases. In a lung virome study, detection of CMV in sputum samples was associated with asthma severity and lung function deficits.^1^ Similarly, seropositivity for CMV was linked to immune and inflammatory responses among patients with chronic obstructive pulmonary disease (COPD),^2^ disease progression in patients with cystic fibrosis,^3^ and airflow limitation (the hallmark of COPD)^6^ among US veterans.^4^ In line with these observations, in a previous study, we found that CMV serology in younger adults (<55 years) predicted risk for COPD‐related mortality in the population‐based cohort of the Tucson Epidemiological Study of Airway Obstructive Disease (TESAOD).^5^ Individuals can develop COPD as a consequence of either lung function deficits that are established in young adult life or an accelerated decline of lung function in adulthood.^7^ It is unknown through which of these trajectories CMV may affect the risk of COPD. In this study, we sought to determine the relation of CMV serology to levels and decline of lung function from young adult life onward in the TESAOD cohort.

## 2 METHODS

TESAOD is a population‐based prospective cohort study of non‐Hispanic white households initiated in Tucson, Arizona in 1972.^8^ At baseline and in up to 11 subsequent surveys done approximately every 2 years over 24 years, participants completed standardized questionnaires on respiratory health and lung function tests. At enrollment, research nurses performed blood withdrawal.

Overall, 403 participants aged 28–55 years at enrollment had CMV data and at least one lung function test from the enrollment and/or follow‐up surveys. CMV serology results at enrollment were calculated as a ratio of the median fluorescence intensity (MFI) of CMV‐antigen‐coupled microspheres and the MFI of negative control microspheres tested at the Myriad‐RBM laboratory (Austin, TX, USA) using the Human MAP infectious panel. Although this infection panel included measurements of antibodies directed against other bacteria and viruses, a priori we included only CMV in this study because of the mounting evidence^1^, ^2^, ^3^, ^4^, ^5^ in support of its involvement in obstructive lung diseases and because of the unique infectious cycle of this virus with periodic reactivations possibly leading to immunosuppressive and pro‐inflammatory effects. CMV serology was validated with the Serion ELISA classic CMV IgG (QED Bioscience Inc., San Diego, CA, USA) on 64 randomly selected serum samples (ρ = 0.64, *p* < 0.0001). Consistent with previous work on this cohort,^5^ we classified participants into CMV serology tertiles (low, medium, and high) that were generated across all adult ages. Serum C‐reactive protein (CRP) levels at enrollment were measured as a marker of systemic inflammation with the enzymatic solid‐phase chemiluminescent immunometric assay performed on the Immulite 2000 (Siemens Diagnostics, Tarrytown, New York).

We used multivariate random coefficients models (with subject and subject‐by‐age as random effects to account for serial correlation) to test the relation of CMV serology at enrollment to lung function levels and decline during adult life. Models were run separately for FEV_1_, FVC, and FEV_1_/FVC as the dependent variables. Models included interaction terms between tertiles of CMV serology and age to test differences in decline. Additional a priori covariates included sex, level of education, CRP serum levels, and body mass index categories at enrollment as well as time‐varying height, smoking status, and pack‐years. Secondary analyses included similar models, but testing interaction terms with years of follow‐up instead of age and including age at enrollment as an additional fixed covariate. To evaluate combined effects of CMV serology and smoking, a four‐group variable based on the combination of CMV serology (low‐medium vs. high) and smoking status (never vs. ever) was generated and tested in random coefficients models.

## 3 RESULTS

Baseline characteristics of the 403 participants included in the present study are shown in Table 1. High CMV serology was positively associated with older age, female sex, lower education level, and higher serum CRP (data not shown).

**TABLE 1:** Characteristics at baseline of the 403 TESAOD participants included in this study.

Age in years: mean (*SD*)	42.3 (8.3)
Sex: females *N* (%)	230 (57.1)
Education level: ≥12 years *N* (%)	344 (85.4)
Body mass index category: *N* (%)	*N* = 388
Underweight	7 (1.80)
Normal weight	224 (57.7)
Overweight	134 (34.5)
Obese	23 (5.93)
Smoking status: *N* (%)	*N* = 402
Never	124 (30.9)
Former	106 (26.4)
Current	172 (42.8)
Pack‐years^a^: mean (*SD*)	22.8 (17.6)
C‐reactive protein in mg/L: geometric mean	1.53
FEV_1_% predicted: mean (*SD*)	95.3 (17.0)
FVC % predicted: mean (*SD*)	98.3 (15.7)
FEV_1_/FVC ratio: mean (*SD*)	80.5 (8.4)
Airflow limitation^b^: *N* (%)	*N* = 403
No airflow limitation	366 (90.8)
Stage 1	15 (3.72)
Stage 2	15 (3.72)
Stage 3 + 4	7 (1.74)
CMV serology: *N* (%)	*N* = 403
Low	176 (43.7)
Medium	124 (30.8)
High	103 (25.6)

^a^ Among smokers.

^b^ Airflow limitation stage 1: FEV_1_/FVC <70% plus FEV_1_% predicted ≥80%; Stage 2: FEV1/FVC <70% plus FEV1% predicted ≥50% and <80%; Stage 3–4: FEV1/FVC <70% plus FEV1% predicted <50%.

**FIGURE 1:**
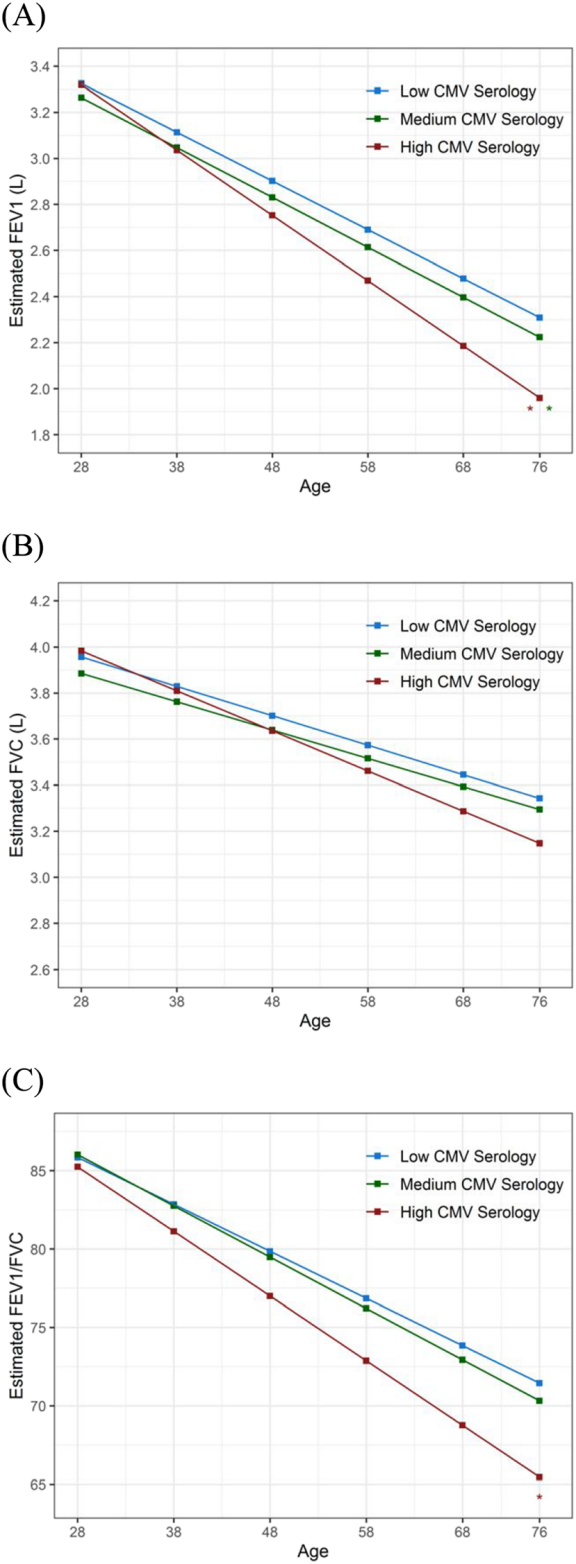
Levels and decline of (A) FEV_1_, (B) FVC, and (C) FEV_1_/FVC ratio in adult life in TESAOD participants across tertiles of CMV serology at baseline. ** FEV_1_ decline significantly steeper for the high CMV serology group than for the low and medium CMV serology groups. * FEV_1_/FVC decline significantly steeper for the high CMV serology group than for the low CMV serology group. Data are estimated from fully adjusted random coefficients models including 403 participants with multiple lung function tests for a total of 2908 observations. Models included interaction terms between CMV serology tertiles and age to test differences in decline and they were further adjusted for sex, height, body mass index categories at enrollment, level of education, smoking status, pack‐years, and serum CRP at enrollment. Lines represent predicted values for a 170‐cm tall female with geometric mean CRP levels of 1.35 mg/L at baseline and ≥12 years of education. CMV, cytomegalovirus; TESAOD, Tucson Epidemiological Study of Airway Obstructive Disease; FEV_1_, forced expiratory volume in 1 s; FVC, forced vital capacity.

During follow‐up, the 403 participants completed on average 7.2 lung function tests per subject for a total of 2908 observations over a mean period of 14.7 years (*SD*: 7 years). In random coefficients models, after full adjustment, high CMV serology at enrollment was associated with a faster decline of lung function during follow‐up. Subjects in the highest CMV serology tertile had faster declines of FEV_1_ and FEV_1_/FVC compared with subjects in the lowest tertile (by −7.9 ml/year, 95% confidence interval CI [−13.9 ml/year, −1.93 ml/year], *p* = 0.010; and by −0.13%/year [−0.23%/year, −0.026%/year], *p* = 0.014; respectively). They also had faster FEV_1_ decline than subjects with medium CMV serology (by −7.6 ml/year [−14.1 ml/year, −1.16 ml/year], *p* = 0.021). No significant associations were found between CMV serology and FVC decline. As shown in Figure 1, results from random coefficients models across adult life indicated that participants with high CMV serology had steeper declines of FEV_1_ and FEV_1_/FVC during adulthood. Despite starting from similar levels in young adult life, by age 75 (the oldest observation was at age 76 years) the estimated FEV_1_ levels for individuals with high CMV serology were 342 ml (129 ml, 556 ml; *p* = 0.002) and 258 ml (33 ml, 482 ml; *p* = 0.025) lower than those of individuals with low and medium CMV serology, respectively.

Because smoking is one of the strongest determinants of accelerated decline of lung function, next we tested whether the above effects of CMV on FEV_1_ and FEV_1_/FVC decline were additive with those of cigarette smoking. As shown in Table 2, estimates from fully adjusted random coefficients models for the four groups generated by the combination of CMV serology and smoking indicated that participants with both high CMV serology and smoking had the steepest decline of lung function. As compared with nonsmokers with low‐medium CMV serology, smokers with high CMV serology had faster declines of both FEV_1_ by −10 ml/year (*p* = 0.004) and FEV_1_/FVC by −0.16%/year (*p* = 0.006).

**TABLE 2:** Estimated decline of FEV_1_ and FEV_1_/FVC from random coefficients models for the four groups generated by the combination of CMV serology at enrollment (low‐medium vs. high) and smoking status (never vs. ever).

	Estimated decline of FEV_1_ (95% CI)	Estimated differences in decline of FEV_1_ (95% CI); *p*
Low‐medium CMV/never smk	−19.5 ml/year (−23.9, −15.2)	Ref
Low‐medium CMV/ever smk	−23.6 ml/year (−27.1, −20.1)	−4.1 ml/year (−9.6, 1.5); 0.152
High CMV/never smk	−27.2 ml/year (−38.8, −15.6)	−7.7 ml/year (−20.1, 4.7); 0.223
High CMV/ever smk	−29.9 ml/year (−35.3, −24.4)	−10.3 ml/year (−17.3, −3.4); 0.004

	Estimated decline of FEV_1_/FVC (95% CI)	Estimated differences in decline of FEV_1_/FVC (95% CI); *p*
Low‐medium CMV/never smk	−0.24%/year (−0.32, −0.17)	Ref
Low‐medium CMV/ever smk	−0.33%/year (−0.38, −0.27)	−0.08%/year (−0.17, 0.01); 0.084
High CMV/never smk	−0.33%/year (−0.52, −0.13)	−0.08%/year (−0.29, 0.12); 0.432
High CMV/ever smk	−0.41%/year (−0.50, −0.32)	−0.16%/year (−0.28, −0.05); 0.006

*Note*: *N*s of subjects and lung function observations per group. Low‐medium CMV/never smk: *N* sbjs = 92; *N* obs = 738. Low‐medium cytomegalovirus (CMV)/ever smk *N* sbjs = 208; *N* obs = 1541. High CMV/never smk *N* sbjs = 19; *N* obs = 115. High CMV/ever smk N sbjs = 84; N obs = 514. Data are estimated from fully adjusted random coefficients models including 403 participants with multiple lung function tests for a total of 2908 observations. The main independent variable CMV/smk was generated by the combination of CMV serology (low‐medium vs. high) and smoking (never vs. ever). Models included interaction terms between CMV/smk and age to test differences in decline and they were further adjusted for sex, height, level of education, pack‐years, and serum CRP at enrollment.

## 4 DISCUSSION

Our findings indicate that high CMV serology in young to mid‐adult life (age 28–55) predicts a subsequent accelerated decline of FEV_1_ (and, in turn, of FEV_1_/FVC). No association was found for FVC, possibly showing that these CMV effects are specific to airflow limitation, the hallmark of COPD.

CMV serology was associated with sex, age, systemic inflammation, and educational level. Although the possibility of residual confounding by additional unmeasured factors (such as occupational exposures) cannot be ruled out, our results on the association between CMV and decline of lung function held true after adjustment for all the above covariates.

One possible interpretation of these results is that high CMV seropositivity in young to mid‐adult life affects the risk for COPD through an increased susceptibility to environmental triggers of accelerated decline of lung function. In line with this scenario is the observation that plasma levels of CMV reactive antibodies are higher in smokers with COPD than controls.^2^, ^4^ In addition, in this analysis, we found that smokers with high CMV serology had the steepest decline of lung function during adult life, although our study was not powered to test possible CMV‐by‐smoking interactions.

In addition to direct effects on the lung,^9^ CMV reactivation can have immunosuppressive effects, increase susceptibility to infections,^10^ and enhance inflammatory responses,^11^ all of which are mechanisms possibly involved in the inception and progression of COPD.

In conclusion, in a long‐term population‐based cohort, we found that CMV serology in young to mid‐adult life is associated with an accelerated decline of FEV_1_ and FEV_1_/FVC during adult life. Future studies will need to address the nature of the association between CMV infection and lung function decline and the possible impact of reducing CMV reactivation on the natural history of COPD.

## AUTHORS CONTRIBUTIONS

Conceptualization: RN, MH, and SG. Statistical analyses: RN, JZ, and DLS. Molecular assays: AS and MH. Lung function data: RN, DLS, FDM, and SG. Writing: RN and SG. Review and editing: all authors.

## CONFLICT OF INTEREST STATEMENT

The authors report receiving NIH award grants made to the University of Arizona.

## ETHICS STATEMENT

The study was approved by the University of Arizona Institutional Review Board.

## Data Availability

The data that support the findings of this study are available on request from the corresponding author. The data are not publicly available due to privacy or ethical restrictions.
